# Using a Black Undergraduate Women Leader Identity Model in an Anti‐DEI Landscape

**DOI:** 10.1002/yd.20670

**Published:** 2025-06-04

**Authors:** Rebecca “Becka” Shetty

**Affiliations:** ^1^ The University of Texas Arlington Texas USA

## Abstract

In a climate where anti‐diversity, equity, and inclusion (DEI) legislation bars leadership educators from using certain terms, sources of knowledge, and overtly liberatory pedagogy, leadership educators must still find a way to meet the needs of diverse student populations. This article explores the use of design thinking in serving Black women students in college. The Black undergraduate women leader identity (BUWLI) model is used to inform the design thinking process and provides educators with ideas for generating educational strategies within the limits of anti‐DEI policy.

## 1 Introduction

Since 2023, 86 legislative bills opposing diversity, equity, and inclusion (DEI) efforts have been introduced in the US Congress across 28 states. Fourteen of these have become law, affecting 12 states across the nation (Chronicle Staff 2024). The effect on DEI work is not limited to a singular region of the country and demonstrates a potential impact on colleges and universities nationwide. These anti‐DEI bills affect higher education through restricting the existence of DEI‐specific offices and staff, mandatory DEI trainings, DEI statements, and identity‐based considerations used in hiring and admissions practices (Chronicle Staff 2024).

Educators like us are still called to support students, even when systemic policies make the work seemingly insurmountable. In this article, I explore design thinking as a framework for serving Black women populations while continuing to enhance the learning of all students within leadership education programs. I explain how the Black undergraduate women leader identity (BUWLI) development model can be used to inform each stage of the design thinking process in developing educational experiences for Black women in colleges, even where anti‐DEI legislation favors racially neutral programming. The BUWLI model serves as a tool for keeping a learner‐centered mindset in leadership education.

## 2 Positionality

I am a mid‐level student affairs practitioner in leadership education, a scholar, and a researcher. I identify as a White woman with Mexican heritage, cisgender, straight, middle‐class, educated, able‐bodied, and Christian. I am not a Black woman; therefore, I cannot directly understand the experience of gendered racism (Davis and Maldonado 2015; Spates et al. 2020; Moody et al. 2022). I do, however, know what it is like to hold privilege, and the goal of my research and of this article is to amplify the experiences of Black women.

## 3 Design Thinking

The emphasis of design thinking is meeting user needs and ensuring an experience that can be evaluated with the user at center of mind (Dam 2024). Within the education profession, we can consider the user our students or learners (Allen and Juarez 2024). Understanding the learner is an essential aspect of liberatory and effective pedagogy. As Daniels and Perkins (2024, 40) reflected, “…leadership educators must first understand who they are teaching so that they can utilize the most appropriate teaching and assessment strategies to foster leadership knowledge.” Varying resources affirm that five stages make up the process of design thinking including: (1) empathize, (2) define, (3) ideate, (4) prototype, and (5) test (Dam 2024; Murtell 2021). In this article, I combine *empathize* with *define*, as well as *prototype* with *test*.

## 4 The BUWLI Model

The Black undergraduate women leader identity development model (Shetty 2020) explains the journey 18‐ through 24‐year‐old Black women in college take toward subsuming *leader* as part of their identity. The model was created using grounded theory methodology (Charmaz 2006; Glaser and Strauss 1967) through interviewing 11 women from two institutions of higher education in the southeast region of the United States.

**FIGURE 1:**
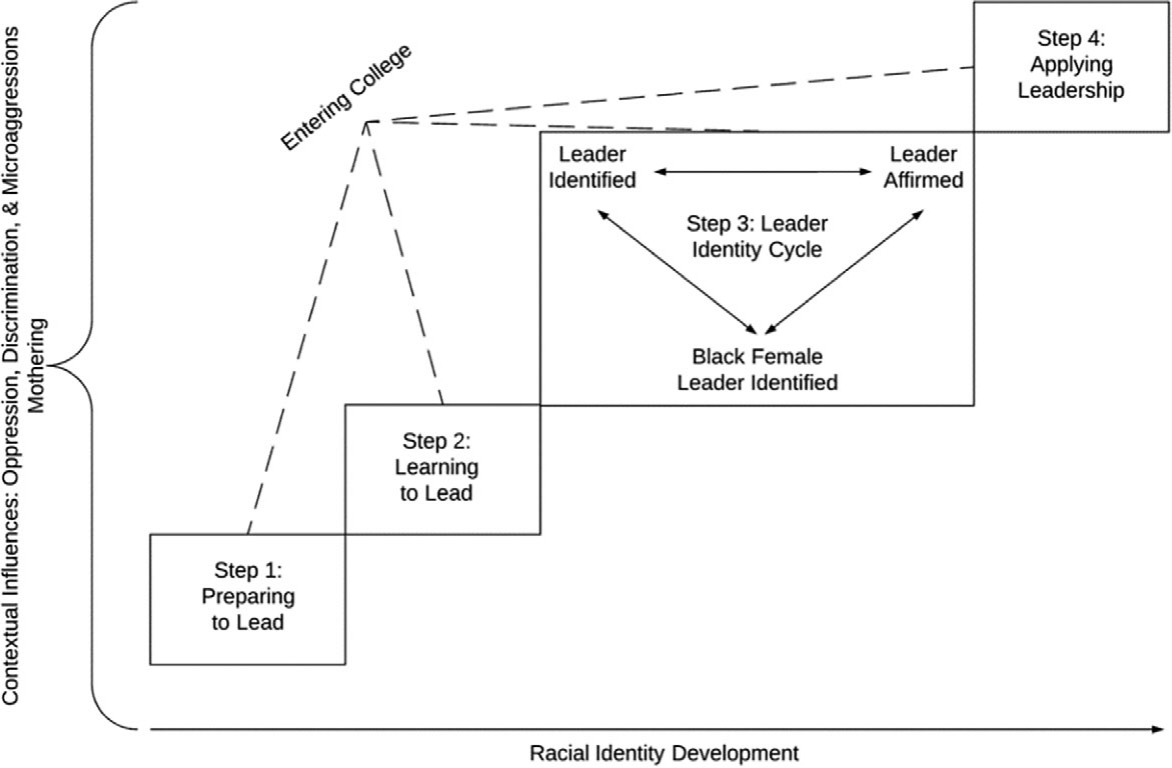
Black undergraduate women leader identity model (BUWLI Model). *Source*: Shetty (2020, 102).

The BUWLI model includes four distinct steps that Black women experience as they develop an identity as a leader (see Figure 1). In Step 1: *Preparing to Lead*, individuals cognitively process the existence of leadership and experience empowerment. Individuals are often influenced by family through instilled values and lessons, such as self‐love and knowing that anything is possible. Step 2: *Learning to Lead* involves individuals better conceptualizing leadership, practicing leadership, and learning more about themselves as leaders in relation to others. Individuals may learn these lessons through formal leadership roles or experiences.

Step 3: *Leader Identity Cycle* includes three codependent and cyclical concepts to emphasize the importance of individuals identifying as a leader, identifying as a *Black female* leader, and being affirmed in these identities. For Black women in college, being a leader is dependent on the external validation and affirmation by others. Although individuals may possess pre‐existing confidence, this is only increased by hearing from others that their leadership practices are effective. In addition, for Black women, a leader's identity is incomplete without understanding how this intersects with their racial and gender identities. Leader identity is directly related to *Black female* leader identity. Step 4: *Applying Leadership* refers to individuals seeking to use their leadership and the power afforded to them to contribute to their communities or give back to others. This final step includes empowering others to seek leadership or helping others overcome obstacles on their way to success (Shetty 2020).

In addition to these steps, the model includes three additional elements: *contextual influences* (oppression, discrimination, and microaggressions; and mothering), *racial identity development*, and a *collegiate focus*. Each of these elements points toward important environmental and sociological factors affecting leader identity development (Shetty 2020).

The BUWLI model visually represents a developmental process in which individuals move from one step to the next, moving upward. However, not every individual will move through these steps cleanly and may experience steps concurrently. With that said, a step‐based model was chosen for its metaphorical representation of the striving many Black women experience on their leadership journey.

One of the most obvious applications for this model is its use within Black women‐specific programming in which cohorts of Black women can grow, fellowship, and thrive together. However, in an anti‐DEI landscape in which states are no longer able to offer programs for singular races, ethnicities, or genders, how does one employ this model in a useful way? The remainder of this article explores the use of the BUWLI model within a design thinking framework.

## 5 Design Thinking: Empathize and Define

Within the design thinking process, “Empathy is crucial to problem solving…as it allows design thinkers to set aside their own assumptions about the world and gain real insight into users and their needs” (Dam 2024). The research and interviews supporting the BUWLI model allow us to empathize with the challenges faced by Black women. The research also helps define the problem for Black women pursuing leadership.

### 5.1 BUWLI Contextual Element: Oppression, Microaggressions, and Discrimination

Existing research demonstrates how Black women in college endure violence and need more support to survive and thrive. A study by Patton et al. (2023) investigated 42 cases of institutional violence against Black women at colleges and universities. The study found common themes among these cases that ultimately represent the ways violence emerges, including betrayal in the form of institutions denying wrongdoing, hate speech, delayed response to hate speech and hate crimes, and institutional disownment when Black students come forward with concerns.

West et al. (2021) summarized the experience of Black college women in academia by describing the inequitable treatment, discrimination, and othering by peers, faculty, and administrators. In a meta‐analysis of 38 articles on Black women's success in college, Porter and Byrd (2021) described the lack of belonging Black women in college feel, particularly at primarily White institutions. Their study also describes the lack of institutional support provided to Black women students, the lack of representation across majors, particularly in STEM fields, and the inability to engage in meaningful identity development without proper support (Porter and Byrd 2021).

The BUWLI model supports and is supported by the aforementioned research. The participants of the study (Shetty 2020) referenced the ways in which they experienced racism and experiences with microaggressions and discrimination. A powerful example was provided by participant Arya. She stated:

I was the only African American female in my AP Chemistry class. And we're in teams. And I had one of my fellow classmates prefer to switch me out with a White male because

he thought that he would be much more beneficial and additional to their team…(Shetty 2020, 91)

Elizabeth reflected on her positionality in a world that was not made for Black female leadership:

…when you notice you're the only person in the room of a certain skin tone that's when

you really realize who you are, and you're trying to wrestle with if you're a monster, or if

you're supposed to belong here, or if you're an invasion of space…I both realized who I represented and also who was not present in the room. (Shetty 2020, 81)

### 5.2 BUWLI Contextual Element: Mothers as Examples of Leadership

The *motherline* is a critical concept rooted in African tradition that has been carried through the centuries. The motherline uses various verbal teachings as a pedagogical tool to shape and raise future generations (King and Ferguson 2011, 67). Dozier‐Henry (2011) identified the role Black mothers play in their children's lives by setting an example and inspiring their children to be the best versions of themselves. Black children begin to associate leadership with the teachings and behaviors observed in their mothers or other women role models in their lives. Black girls learn how to be Black women, assuming various roles such as friend, sister, mother, and daughter, from their mothers (Dozier‐Henry 2011; Sakho 2017).

King and Ferguson (2011) described the communal leadership taught through the many female relationships in a young Black woman's life. Black women receive lessons of leadership from their mothers and may also have a complex web of female figures in their community from whom to learn. In the case of Black women students, mentors, faculty, and staff at their institution may serve in these roles when they are away from home and their primary caretakers (Brown and Mendenhall 2023; Fields and Valandra 2024).

In the research on the BUWLI model, Arya described messages she heard about leadership from her mother: “…one thing that my mother kind of like grilled in me when I was little was that you have a voice, and don't be afraid to use it” (Shetty 2020, 92). Similarly, Linda heard messages from her mother about surviving within current societal norms: “…something that my mom always told me…that I have to work twice as hard or three times as hard as someone as someone who was White because of me being Black and also being a woman…” (Shetty 2020, 82). Black women appear to grow and thrive in spaces where they learn from mothers and important mentors, and reflect on lessons learned from these individuals. These lessons build efficacy, capacity, and identity essential for the leadership learning journey (Guthrie and Jenkins 2018).

### 5.3 BUWLI Step 3: Leader Identity Cycle

Affirmation plays an essential role within Step 3 of the BUWLI model. Affirmation allows Black women in college to connect the dots between their identity as a leader and as a Black woman leader capable, able, and worthy of leadership (Shetty 2020). Recognizing how race and gender alters the course of leadership identity reinforces the importance of individualizing the attention given to our Black women students. One way we can assist is through affirmation. Alyssa, a BUWLI participant, confirmed this sentiment when she said:

…once again reassured by those people that you're leading that you are doing a great job and that you should even try to be a leader of something…because if other people see me as a leader…I also see myself as a leader. (Shetty 2020, 112)

Similarly, Jade was able to describe the heart of Step 3 when she said:

I think it was like words of affirmation and encouragement from others. And also um

being aware that my identity is typically not seen in a leadership role. And so being a

Black woman and calling yourself a leader and making other people aware of that is just so powerful. (Shetty 2020, 113)

### 5.4 Defining the Problem

It is not simple to cleanly define the problem surrounding Black women and the leadership development journey in college. Their overall experience in college may be rife with difficulty, and this difficulty may be translated to their experiences with leadership. The challenges may be unique to each individual and dependent on which institution they attend and the region of the country in which they reside. Key problems related to Black women in college include: (1) the lack of space to unpack their identities (Porter and Byrd 2021), (2) feeling unsupported in their development as individuals and as leaders, and (3) the need for institutions to meet students where they are by listening, understanding, and allying with Black women.

## 6 Design Thinking: Ideate

According to Dam (2024), ideation is a chance to “…look at the problem from different perspectives and ideate innovative solutions to your problem statement” (para 10). For the sake of this article and understanding how the ideation phase reflects the uniqueness of each institutional context, I provide general insight and guidance on ideation considerations important for Black women as learners.

When we look at the BUWLI model, we see leadership learned and conceptualized in several ways. To develop their leadership identity, Black women must experience leadership knowledge (Steps 1 and 2), leadership training (Steps 2, 3, and 4), leadership development (Steps 1, 2, 3, and 4), and leadership observation (Steps 1, 2, and 3). These four ways of approaching leadership learning meet various learning styles and provide holistic development for the learner (Guthrie and Jenkins 2018). Ideation must include brainstorming a range of exercises, activities, and programs that meet all these pedagogical needs. Activities should include watching, doing, reflecting, and practicing. As each practitioner seeks to ideate for their respective populations, the encouragement is to ensure various learning styles and best practices are being used while consulting the learner in mind.

### 6.1 Culturally Relevant Pedagogy

In addition to engaging a range of learning styles and ensuring holistic educational design, ideas generated should be mindful of the cultural needs of Black women. I suggest referencing *Operationalizing Culturally Relevant Leadership Learning* (Beatty and Guthrie 2021) for more on applying theory to practice. In this text, Beatty and Guthrie (2021) encouraged educators to consider the diversity of learners that contribute to the educational space and how acknowledging systems of power can inform student efficacy, capacity, and identity. Keep in mind how, in anti‐DEI environments, educators may not be able to use language around power, oppression, and identity. However, the use of culturally relevant pedagogy may still *inform* the ideation process, allowing us to determine the final educational approach that we can then modify to be legally compliant.

### 6.2 BUWLI Step 4: Applying Leadership

Constructivism and co‐constructivism in leadership learning and education allow us to see both educators *and* students as creators of leadership knowledge and effective practice (Chunoo et al. 2019). When we reference the BUWLI model, we see the potential for Black women to effectively apply leadership through empowering others. Tina, a study participant, describes her experience:

…‘cause I'm always like tryin’ to get my friends like you should try this. Like,

you should try, you know, being on this exec board, you should try doin’ that just

because, like, if I can do it, you know you can do it too. (Shetty 2020, 114)

As they have gone through the leader identity development steps of learning to lead, becoming a leader, and applying their leadership skills, they, in turn, can become co‐creators in the mentorship, methods, and strategies for leadership. It would behoove practitioners to include the voices of students when ideating how best to teach and explore leadership through focus groups, student advisory boards, or student employment as experiential learning.

## 7 Design Thinking: Prototype and Test

When thinking of recommendations for practice, we can return to the areas that were identified from the empathize and define phase of design thinking. Although trying to empathize, several themes emerged, including understanding oppressive systems, the role of mothers and mentors, and the importance of affirmation for Black female leaders. Black women students may have trouble examining their identities, finding appropriate developmental support, and being met where they are in their experiences as marginalized women.

Programs designed for all students *can* still target the concepts supporting the leadership development of Black women (e.g., the socialization of leadership, the role of family and mentors, and the effect of life experiences on leader confidence). Since students can serve as co‐creators of knowledge, asking questions and creating opportunities for reflection can help Black women conceptualize leadership in a way that is authentic and helpful to them (Volpe White et al. 2019) even when couched in more generalized educational practices. Below are some possible exercises that can address the specific concerns of Black female leadership learners while also benefiting other students.

### 7.1 Socialization of Leadership

A key issue identified through the design thinking process was Black women not having the space to process their identities. Varying childhood development and socialization theories describe how influences all around us consistently shape who we are and what we believe about the world (Bronfenbrenner 1979; Harro 2010; Piaget 1936). The challenge to our socialization is that sometimes we begin to believe untruths about ourselves, the world around us, and who we may be as leaders. As educators, we can encourage students to seek spaces to unpack their life experiences while also creating those spaces ourselves.

First, in an anti‐DEI landscape, encouraging students to find spaces with those who share their identities is still important. Currently, many laws do not ban student organizations, so educators should stay informed on the race and gender‐based organizations on their campus that continue to function. Similarly, some states do not ban content within the classroom or academic‐based programming that focuses on race or gender, though this may change in the future. Encourage students to take courses or collaborate with race or gender‐based academic centers to provide racial and gender affinity. These spaces will allow students to more authentically reflect on their experiences with peers and faculty who are still permitted to be open about the role identities, power, and privilege play in their lives and in leadership. Individual advising and mentor relationships also provide space for students to process their experiences. Educators can use individual conversations and advising sessions to ask students how they are processing national and global current events and provide support depending on the response.

Within the context of extra and co‐curricular programming, leadership educators can still host exercises that allow students to reflect and hear from others about personal backgrounds and lived experiences that inform the leadership journey. This allows our Black women students to explore and reflect on Step 1 of the BUWLI model, preparing to lead. Exercises that may benefit our Black women students include personal histories, life timelines, milestone moments reflections, and life mapping (Volpe White et al. 2019). This allows student to introspect on how their backgrounds and experiences have shaped them and their leadership. Reflection questions for these types of exercises may include:

- Which life experiences have most shaped who you are as a person and as a leader?
- What is one thing you love about yourself that informs who you are as a leader?
- What are the barriers you have faced on your leadership journey, and how have you overcome them?
- Who has helped you on your leadership journey?

Educators should employ reflective listening skills and follow up with questions and phrases such as “tell me more about that,” “how?” and “why?” These probes encourage students to introspect further without asking explicitly about identities or experiences with power or privilege.

### 7.2 Role of Family and Mentors

Contreras (2023, 109) wrote specifically about familial influences for women of color: “Families within Communities of Color are rich with stories, experiences, and lessons of caretaking, survival, and coping that impact leadership identity development, and they should be acknowledged.” The role of family members and mentors in leadership education and identity development is not unique to the BUWLI model, even if *mothering* and the role of mother figures is more specific to the African American community. The original leadership identity development model (LID) (Komives et al. 2005) also includes adult influences.

Activities involving active reflection on lessons learned from mothers, caretakers, and mentors may help students continue to form their leadership identity and self‐concept as a leader. First, practitioners can integrate values exploration activities such as a values auction, life mapping, “trash your values,” the free VIA Institute on Character strengths assessment (Peterson and Seligman 2004), birthday speech writing, reflective narratives, or art projects. By allowing students to identify values and lessons taken from their childhood and memories, they can reflect on the ways in which their caretakers and mentors contributed to their understanding of leadership. I suggest encouraging discussion among students to learn from each other on which values are most important to them and why. Points of dialogue could include:

- Who instilled the values you have today?
- What role did your mother, father, or other caretaker play in how you understand your values and leadership?
- How would you like to emulate or not emulate your caretakers when practicing leadership?
- How does your mother, father, or other caretaker continue to support your leadership journey?

The addition of father to these reflection questions includes students who may have also learned from their fathers, though it keeps the theme of mothering and the motherline intact for our Black women students. Note how none of these questions violate anti‐DEI legislation, yet still allow our Black women students to connect to pieces of their identity that influence their leadership learning and journey.

In addition, educators can create structures for students to pursue mentorship or connect regularly with mothers and caretakers. Assignments may include creating conversation guides for student and caretaker or mentor, asking how leadership plays a role in the lives of those adult mentors, and asking for guidance or advice. By connecting with a trusted family member or mentor, students can continue to receive more authentic support to help in their developmental journey that is not stifled by the inability to discuss how race and gender influence life and leadership.

### 7.3 Affirmation

It is possible for Black women to fail to identify themselves as leaders and refuse the title of *leader* (King and Ferguson 2011). Because of this, I recommend the use of strengths or skills evaluations to help students reflect on the ways they positively contribute to their communities as leaders. Potential assessments include the Student Leadership Practices Inventory (Kouzes and Posner 2025), Emotionally Intelligent Leadership for Students: Inventory (Shankman et al. 2015), CliftonStrengths for Students assessment (Gallup 1999), or the Student Leadership Competencies Inventory (Seemiller n.d.).

Building leadership efficacy is an essential element of the leadership learning journey, and helping students identify their areas of strengths can help them in their growth toward a leader identity (Devies 2024; Guthrie et al. 2021). To further help with external affirmation needed for Black women in their leadership identity development journey, I recommend 360‐degree feedback opportunities, group affirmation exercises, and intentional feedback from faculty or staff mentors or advisors. These types of exercises allow Black women to see how they contribute positively to their groups and help identify specific strengths of which they may not be aware (Contreras et al. 2024).

Continued reflection is critical to helping solidify confidence and increase leadership identity. Although this is one small step, asking and recognizing the efficacy and confidence development in our Black women students will shape the programming efforts to ensure we are meeting students where they are in their journey. Potential conversation starters or discussion questions for these assessments and 360‐degree feedback include:

- How have you seen your strengths at play in your daily life?
- How can your strengths help you succeed as a leader?
- Wow, can you use your strengths to overcome leadership challenges?
- How does it feel to receive feedback from others regarding your ability as a leader?
- What is one element of the feedback that serves as encouragement to you and your abilities?

The programmatic suggestions above are simply suggestions. The step after prototyping is testing and ensuring you are meeting learner needs and allowing students to thrive. Anti‐DEI legislation typically does not prevent one‐on‐one conversations with students to discuss their well‐being and success. I encourage educators to talk with their Black women students before, during, and after programming to see if the programs are allowing them to grow as leaders per the knowledge gleaned from the BUWLI model and other literature. With a design thinking mindset, the beauty of prototyping is that perfection is not expected. If any of the ideas generated do not produce desired results, new ideas can be enacted and assessed again.

On a larger scale, data‐driven approaches can help enhance this type of programming even within an anti‐DEI landscape. Educators can compare retention and graduation rates of students in their programs against the student body. Pre‐ and post‐tests that assess leadership skills and competencies or the application of learning can demonstrate the value of leadership education programs and determine whether all demographics are being served well.

### 7.4 Programming Example: University of Texas at Arlington Fall Leadership Retreat

The Fall Leadership Retreat (FLR) at the University of Texas at Arlington provides learning in all three areas outlined above, including the socialization of leadership, analyzing the role of family and mentors, and embedding affirmation, all of which benefit the Black female population. FLR is hosted by the Follett Student Leadership Center and is open to all students, from first year through graduate students. The event is a Friday evening through Sunday morning, off‐campus, with approximately 100 students attending each year.

The curriculum includes seven workshops on the following topics: identifying leadership strengths and growth areas (socialization; affirmation; role of family), integrity and ethical decision making (role of family), emotional intelligence (socialization), creating and sharing a vision (affirmation), communication and collaboration with others (socialization), social responsibility (socialization; affirmation), and making an impact in one's communities (socialization; affirmation). Throughout these sessions, questions like those listed above are asked, encouraging Black women and their peers to consider how they have come to learn about leadership, what values they hold, and how those were developed, and helping them identify their strengths and build confidence.

Regarding compliance and effectiveness of programming, I encourage educators to stay informed on the boundaries of DEI legislation. Create checklists of vocabulary or programming that is prohibited while also maintaining a running list of inclusive terms that can be used, such as “student achievement,” “leadership development,” “student engagement,” “mentorship,” and “institutional affinity.” Similarly, ensure your program learning outcomes or objectives remain neutral, focused on areas such as leadership growth and development, community building, social support, mentorship, and general civic or social responsibility.

The social responsibility and impact sessions were affected by state Senate Bill 17 in Texas prohibits DEI terminology and training. These sessions were edited for compliance. Instead of using words like power, privilege, race, gender, and other DEI‐related examples, we had to edit our content toward neutrality. The facilitator of the social responsibility workshops now asks students to reflect on their influence, places where they are respected and have relationships, places where their voice is heard, and what roles give them the ability to influence others. In this session, students are given a framework for action that includes building relationships, creating common goals with others, gathering and analyzing data for the problem being addressed, and acting collaboratively with others.

They are then asked to brainstorm the people, communities, or issues they are most passionate about and to whom or what they commit to being socially responsible. The impact session focuses on a role‐playing exercise in which students are given varying levels of responsibility (a word changed from “power”) to bestow others with responsibility and the ability to act. It is a metaphor for the ways we can encourage others to be leaders, to take action, and to make a change in the world around them. Students reflect on the roles they had in the exercise, how they chose to act in those roles, and how others may have felt about their use of their responsibility.

In 2024, 87.5% of attendees (we have a 100% response rate as our assessment is built into the retreat) indicated they agreed or strongly agreed the retreat increased their sense of belonging at UTA; 84.37% of attendees agreed or strongly agreed their confidence to lead a group increased. Our population of Black/African‐American identifying students made up 16.6% of the attendees (16 of 96), with 14 being Black women. This is representative of our campus diversity, where 13.4% of students identify as Black/African‐American\. Only six Black women chose to identify themselves in the survey (it had the option for anonymity). Of those six, 5 agreed or strongly agreed that the retreat increased their sense of belonging at UTA, and all 6 agreed or strongly agreed that their confidence in leading a group increased.

## 8 Conclusion

I want to acknowledge how the practices and examples above do not allow us to center the specific language, identities, and diversity of the Black women's populations seen on college campuses. I do not suggest this way of educating and existing is ideal or appropriately responsive to diverse student bodies. In addition, these exercises are often already used in leadership learning settings. I recommend shifting our perspective on the utility of varying educational exercises and dialogue to recognize their power even within marginalized populations and adjusting them to include deeper reflection and more intentional conversations among students.

Leadership educators can attempt to retain the values of justice education, liberatory education, and support for marginalized students (Harper and Kezar 2021; Rocco and Beatty 2023; Wiborg et al. 2023) through educational programs and services. We can change our language to reflect more universally accepted values while still intentionally designing programs allowing marginalized students to flourish. The recommended practices in this article are helpful for all students; they are just particularly helpful for Black women, given the findings of the BUWLI study and model.
